# The longitudinal association of eating behaviour and ADHD symptoms in school age children: a follow-up study in the RHEA cohort

**DOI:** 10.1007/s00787-021-01720-x

**Published:** 2021-02-18

**Authors:** Vasiliki Leventakou, Moritz Herle, Mariza Kampouri, Katerina Margetaki, Marina Vafeiadi, Manolis Kogevinas, Leda Chatzi, Nadia Micali

**Affiliations:** 1 Department of Health Research Governance, Ministry of Public Health, Al Rumaila, PO Box: 42, Doha, Qatar; 2 Department of Social Medicine, Faculty of Medicine, University of Crete, Heraklion, Crete, Greece; 3 Department of Biostatistics and Health Informatics, Institute of Psychiatry, Psychology and Neuroscience, King’s College London, London, UK; 4 Barcelona Institute for Global Health (ISGlobal), Barcelona, Spain; 5 Department of Preventive Medicine, Keck School of Medicine, University of Southern California, Los Angeles, MN USA; 6 Department of Genetics and Cell Biology, Faculty of Health, Medicine and Life Sciences, Maastricht University, Maastricht, Netherlands; 7 Great Ormond Street Insititue of Child Health, University College London, London, UK; 8 Department of Psychiatry, Faculty of Medicine, University of Geneva, CH-1205 Geneva, Switzerland; 9 Departement of Gynecology, Obstetrics and Pediatrics, University of Geneva, CH-1205 Geneva, Switzerland

**Keywords:** Eating behaviour, Longitudinal, ADHD, Childhood

## Abstract

Previous evidence suggests a link between attention deficit hyperactivity disorder (ADHD) symptoms and disordered eating behaviours; however, the direction of the causal association remains unclear. Building on our previous research, we aimed to examine the longitudinal association between eating behaviours at 4 years, ADHD symptoms at 6 years of age, and the role of body mass index (BMI). We included children from the RHEA mother–child cohort in Greece, followed up at 4 and 6 years (*n* = 926). Parents completed the Children’s Eating Behaviour Questionnaire (CEBQ) to assess children’s eating behaviour at 4 years and the ADHD Test (ADHDT) and Child Behaviour Checklist for ages 6–18 (CBCL/6–18) to evaluate ADHD symptoms at 4 and 6 years, respectively, as well as measures of BMI. Longitudinal structural equation modeling (SEM) was carried out to evaluate the associations of all variables between 4 and 6 years. Food responsiveness at 4 years was positively associated with hyperactivity at age 6, whereas emotional overeating was negatively associated with hyperactivity. There was no evidence of an association between eating behaviours of preschoolers and BMI at 6 years, or BMI at 4 years and later ADHD symptoms and vice versa. Findings suggest that food responsiveness is an early marker of ADHD symptoms at 6 years of age. In contrast to our hypothesis there was no significant association between ADHD at age 4 and BMI at age 6.

**Supplementary Information:**

The online version contains supplementary material available at 10.1007/s00787-021-01720-x.

## Introduction

Attention deficit hyperactivity disorder (ADHD) has been identified as one of the most common childhood-onset neurodevelopmental disorders worldwide [1], with symptoms of the disorder (inattention, impulsivity and hyperactivity) persisting into adulthood [2]. Current evidence suggests that children diagnosed with ADHD continue to have clinically significant symptoms of ADHD as adults, in up to 65% of cases, regardless if they meet the full criteria for adult ADHD [2, 3]. Children with ADHD may be predisposed to aberrant eating behaviours, often leading to adverse outcomes such as higher risk for overweight/obesity [4–6]. Increasing evidence from recent systematic reviews and meta-analyses suggests a positive association of core symptoms of ADHD with disordered eating [7, 8]; however, this association has not been sufficiently examined, especially at young ages, and its direction is still not clear. Meta-analysis of neuroimaging studies has shown that there are shared neurobehavioral circuits in ADHD, eating disorder, and obesity in pediatric populations [9]. However, cross-sectional analyses fail to estimate the direction-of-effect between eating behaviours and ADHD symptoms. The few longitudinal studies [10–14] conducted up to date have relied on individuals with clinical ADHD symptoms, thus it is unknown whether these findings can be extrapolated to non-clinical populations. In addition, existing studies have focused on binge-eating behaviours (such as seen in binge eating disorder and bulimia nervosa) mainly in late childhood and onwards, missing to capture a wide range of eating behaviours (e.g., emotional eating, food responsiveness) in early childhood.

Our previous work was the first population-based study that investigated the link between ADHD symptoms and eating behaviours in preschoolers [4, 15]. We observed that food approach eating behaviours such as food responsiveness (urge to eat in response to external cues, such as sight or smell of food) and emotional overeating (overeating as a response to negative emotions) were associated with symptoms of ADHD, specifically attention and behaviour regulation in children aged 4 years. Building on our previous research and in an effort to provide better insight into the potential underlying mechanisms between eating behaviours, ADHD symptoms, and obesity [4, 5, 8, 9], we hereby aimed to examine the longitudinal association between aberrant eating behaviours at 4 years and ADHD symptoms at 6 years analyzing data from the Rhea birth cohort study. Specifically, we hypothesized that food approach behaviours at 4 years would be prospectively associated with ADHD symptoms at 6 years. Moreover, given that ADHD symptoms are associated with higher BMI we also hypothesized that ADHD symptoms at 4 years would be prospectively associated with higher BMI at 6 years. To the best of our knowledge no prior studies have examined this association over time in early childhood.

## Methods

### Study population

Starting in February 2007 and within a 12-month period pregnant women were invited to participate in the Rhea birth cohort study in Crete, Greece, [16]. Women were contacted before the 15th week of gestation and those consented to participate were followed up at various times during pregnancy, at birth and for children’s follow-up at 9, 18 months, and at 4 and 6 years of age. Dietary, environmental and psychosocial exposures were assessed during pregnancy and early childhood with the use of face-to-face, self-administered questionnaires and medical records. The study was approved by the Ethical Committee of the University Hospital of Heraklion (Crete, Greece), and all participants provided written informed consent.

Out of 1,363 singleton live births, 872 children were followed up at 4 years and 604 at 6 years of age. In total, 30 children were excluded due to diagnosis of neurodevelopmental disorder (pervasive developmental disorder), other medical conditions (i.e., plagiocephalus, microcephalus, hydrocephalus and brain tumour) or incomplete assessments. Analyses included all participants with available data for at least one-time point (*n* = 926).

### Eating behaviour at 4 years

Children’s eating behaviour was assessed using the Children’s Eating Behaviour Questionnaire (CEBQ) [17]. Primary caregivers/parents were asked to fill out this questionnaire, referring to their children’s eating style, with the guidance of a dietician. Subscales of this instrument have been previously described in detail [4]. Briefly, the 34-item questionnaire consists of the following subscales: food responsiveness; enjoyment of food; desire to drink; emotional overeating; satiety responsiveness; emotional under eating; slowness in eating, and food fussiness. The original English questionnaire was translated using the standard forward–backward translation method, cognitive debriefing process and pretesting. The food responsiveness and emotional overeating subscales were included in these analyses.

### ADHD symptoms

The assessment of ADHD symptoms at the 4-year follow-up has been previously described in detail [4]. Briefly, the 36-item ADHD questionnaire [18], which is based on the diagnostic and statistical manual of mental disorders (DSM-IV) criteria for ADHD, was completed by the mothers. The test includes three subscales (hyperactivity, inattention and impulsivity) and an index for total ADHD difficulties. The ADHDT has been translated and adapted for the Greek population [19].

The Child Behaviour Checklist for ages 6–18 (CBCL/6–18) was used to assess ADHD symptoms at 6 years of age. CBCL is a 113-items parental questionnaire designed to assess behavioural and emotional difficulties in children 6–18 years of age. The CBCL offers two alternative ways to summarize its items, the empirically-based syndrome scales which include the Attention Problems Scale and the DSM-oriented scales which include the Attention deficit/hyperactivity problems scale. In our analysis we used both scales, since both evaluate ADHD-related symptoms. The CBCL has been translated, adapted and standardized for the Greek population [20].

### Maternal and child characteristics

Maternal age (years), maternal education (low level: ≤ 6 years of school, medium level: 7–12 years of school, high level: university or technical college degree) and maternal origin (Greek, non-Greek) at recruitment or delivery.

Child’s gender at birth (male/female), child’s BMI in categories of underweight/normal, overweight, obese, kg/m^2^) and BMI z-scores as a continuous variable at the age of 4 and 6 years. BMI was calculated from height and weight and BMI z-scores have been developed based on the Rhea cohort-specific gender and age-adjusted growth curves. BMI cut-offs were based on International Obesity Task Force (IOTF) definitions [21]. Prior to analyses, variables included in the model: emotional overeating, food responsiveness, hyperactivity, impulsivity and attention problems, were regressed by the above covariates (child’s sex, maternal age at birth, and maternal education).

### Statistical analyses

A longitudinal structural equation model was fitted. All variables observed at age 4 were allowed to be associated with all observed variables at age 6 years. All predictor variables included in structural equation models were adjusted for each other, such that for example the estimated association between food responsiveness at 4 years and ADHD symptoms at 6 years was adjusted for all other predictors included at 4 years. We assumed data would be missing at random (MAR) and variables associated with potential dropout were included as covariates in the model: maternal age at birth, and maternal education at recruitment. To retain the greatest number of participants, we conducted the estimation using full information maximum likelihood (FIML). After fitting a full model including all variables, subsequent submodel was tested constraining paths to zero. Model fit was compared using standard metrics; log-likelihood ratio test, Akaike’s Information Criterion (AIC) and Bayesian Information Criterion (BIC) with lower values indiating a better model fit. All analyses were conducted in Stata version 16.

## Results

Descriptive statistics of the study population at baseline, 4 years and 6 years are presented in Table 1. Participating mothers had a mean (± SD) age of 29.77 (± 4.99) years at delivery. The majority were of Greek origin (93.9%) and had medium educational level (50.29%). At baseline the study included 444 (47.95%) boys and 482 (52.05%) girls. At age 4 (*n* = 681, 78.10%) and 6 (*n* = 406, 67.22%) most of the children had underweight/healthy weight. A comparison of demographic characteristics of participants at baseline and those who had data at both 4 and 6 years is listed in Supplementary Table 1 (Table S1). Table 2 includes correlations between eating behaviours, ADHD symptoms and BMI z-scores at 4 and 6 years of age. All variables were positively correlated with each other, with correlation coefficients ranging from 0.12 between hyperactivity and zBMI at age 4 and 6–0.89 between hyperactivity and attention problems at age 6.

**Table 1:** Maternal and child characteristics of the study population at each time point

	*N*	% or Mean ± SD
Baseline		
Maternal age (years)	917	29.77 ± 4.99
Maternal origin, *n* (%)		
Greek	862	93.9
Non-Greek	56	6.10
Maternal education, *n* (%)		
Low	146	16.31
Medium	459	51.28
High	388	32.40
Child's sex, *n* (%)		
Male	444	47.95
Female	482	52.05
4-year follow-up		
Child's age (years)	872	4.24 ± 0.24
CEBQ		
Emotional overeating	564	1.68 ± 0.56
Food responsiveness	576	2.14 ± 0.83
ADHD symptoms		
Hyperactivity	650	5.49 ± 5.24
Impulsivity	650	5.16 ± 4.21
Child’s BMI (kg/m^2^), *n* (%)		
Underweight/normal	681	78.10
Overweight	126	14.45
Obese	65	7.45
Child’s BMI z-scores	872	0.13 ± 0.94
6-year follow-up		
Child's age (years)	604	6.57 ± 0.27
CBCL		
Hyperactivity	576	3.41 ± 2.75
Attention problems	573	2.99 ± 2.81
Child’s BMI (kg/m^2^), *n* (%)		
Underweight/normal	406	67.22
Overweight	129	21.36
Obese	69	11.42
Child’s BMI z-scores	599	0.41 ± 0.93

*SD* standard deviation, *CEBQ* child's eating behaviour questionnaire, *BMI* body mass index, *ADHD* attention deficit hyperactivity disorder, *CBCL* child behaviour checklist

**Table 2:** Pairwise correlations among variables at 4 and 6 years of follow-up, *N* range = 375–650

	4 years					6 years		
	FR	EOE	Hyperactivity	Impulsivity	BMI z-scores	Hyperactivity (CBCL)	Attention Problems (CBCL)	BMI z-scores
4 years								
Food responsiveness (FR)	1							
Emotional over-eating (EOE)	0.58*	1						
Hyperactivity	0.17*	0.19*	1					
Impulsivity	0.15*	0.13*	0.72*	1				
BMI z-scores	0.26*	0.15*	0.12*	0.15*	1			
6 years								
Hyperactivity (CBCL)	0.13*	0.01	0.50*	0.44*	0.10	1		
Attention problems (CBCL)	0.12	0.06	0.46*	0.42*	0.08	0.89*	1	
BMI z-scores	0.25*	0.21*	0.12	0.16*	0.82*	0.12*	0.13*	1

*P-value < 0.01

### Structural equation model

**Fig. 1:**
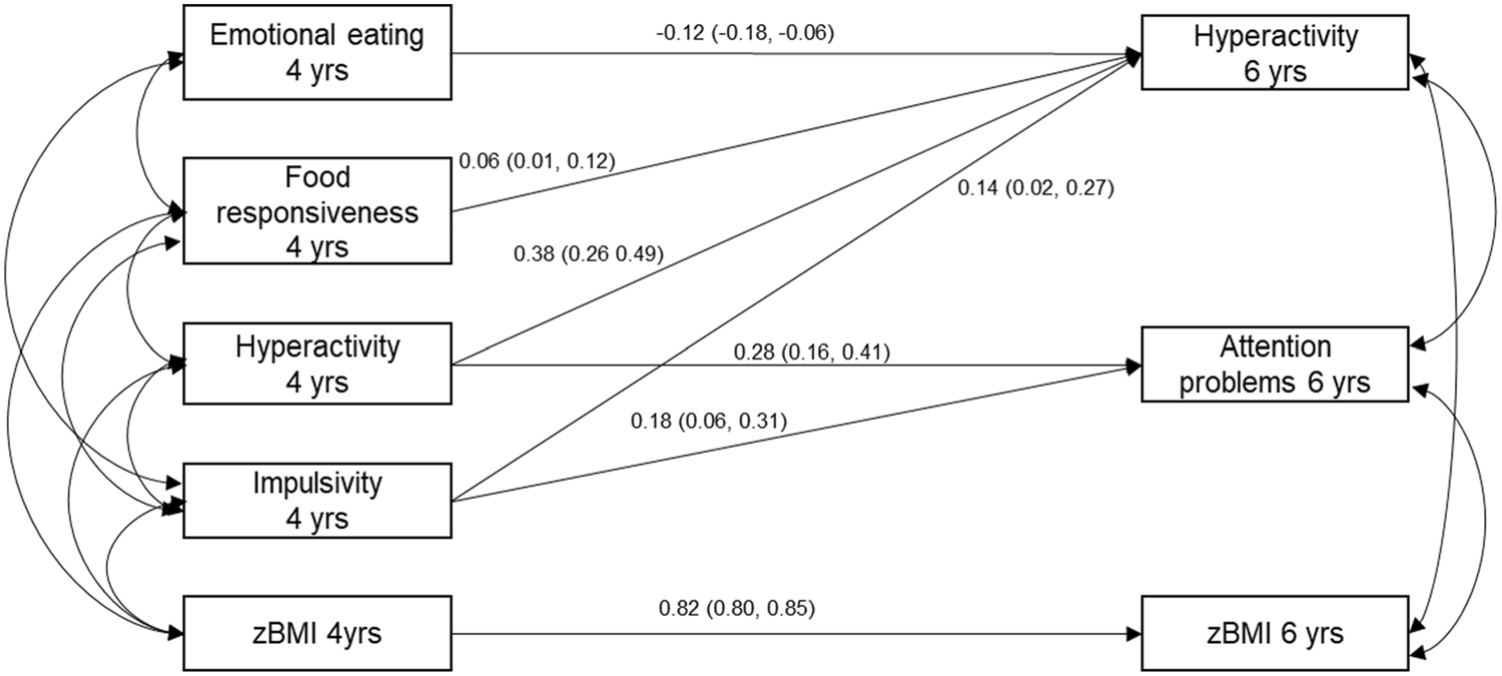
Path diagram of eating behaviours and ADHD symptoms at 4 years, CBCL subscales at 6 years and BMI z-scores at both time points, submodel including only significant paths (*n* = 926). Estimates are not standardized

Results from the full SEM are listed in Table S2, showing standardized beta coefficients, 95% confidence intervals and *p*-values. In a subsequent submodel, all path estimates with 95% confidence intervals crossing zero were dropped from the model to achieve a more parsimonious solution. Path estimates from this submodel were carried forward for interpretation and results are presented in Table S3. This submodel fit the data well, and there was no model fit difference between the full model and submodel [diff Log Likelihood (*df*) = 6.35 (8), *p* = 0.61]. Additional model fit indices, the AIC and BIC confirmed the improved fit of the submodel by assigned lower values (see full fit statistics in Table S4). The submodel is illustrated in Fig. 1. Path estimates from the submodel are illustrated in Fig. 1 (list of estimates in Supplement Tables 2, 3). We found that emotional overeating at 4 years was prospectively associated with lower levels of hyperactivity (*β* = − 0.12, 95% CI − 0.18, − 0.06, *p* < 0.05) but not with attention problems at 6 years. Food responsiveness at 4 years was positively associated with hyperactivity (*β* = 0.06, 95% CI 0.01, 0.12, *p* < 0.05) but not with attention problems at 6 years. As expected ADHD symptoms at 4 years positively contributed to ADHD symptoms at 6 years. Both hyperactivity and impulsivity at 4 years predicted higher hyperactivity (*β* = 0.38; 95% CI 0.26, 0.49 and *β* = 0.14; 95% CI 0.02, 0.27, respectively) and attention problems (*β* = 0.28; 95% CI 0.16, 0.41 and *β* = 0.18; 95% CI 0.06, 0.31, respectively) scores at 6 years. BMI at age 4 was not related with later hyperactivity or attention problems, but tracked substantially (*β* = 0.82; 95% CI 0.80, 0.85, *p* < 0.05) to the age of 6 years. There was no evidence of a longitudinal association between food responsiveness and emotional overeating with BMI at 6 years.

A subsequent step included a sensitivity analysis and the same model was fitted separately for boys and girls. Results differed slightly between the two groups but were overall in the same direction (see Table S5 for a full list of path estimates).

In these sex-stratifed sensitivity analyses, associations seen in whole group analyses remained, but with slight differences between the two groups (Table S5). For boys, food responsiveness was not significantly associated with attention problems at 6 years. Statistical significance was also attenuated between emotional overeating and impulsivity at 4 years and ADHD symptoms at age 6, in the girls group, potentially due to the reduced sample size in the subpopulations.

## Discussion

The present study is the first to explore the longitudinal association between food approach behaviours and ADHD symptoms across childhood. Food responsiveness at 4-year predicted hyperactivity at age 6, whereas this association was negative for emotional overeating. Both ADHD symptoms and BMI tracked longitudinally from age 4–6 years. Contrary to our hypothesis, there was no association between ADHD symptoms at 4 years and later BMI or vice versa. Finally, we did not observe any associations between eating behaviours of preschoolers and BMI at 6 years, as well as no evidence for an association between eating behaviours and later attention problems. When testing boys and girls separately, gender did not affect the observed associations, suggesting that similar risk pathways operate across genders.

Our prospective findings indicated a positive longitudinal association between food responsiveness (urge to eat in response to external cues, such as sight or smell of food) and ADHD symptoms at age 6, even when accounting for their correlation at age 4. In this case, overeating in the form of food responsiveness also known as external eating, might result from a predisposition to impulsivity, which in turn is considered as a central symptom for both ADHD [22] and eating pathology [23], in particular bulimic type behaviours/disorders, as shown in population based studies. Recent twin analyses have suggested that food responsesivenss and reward responsiveness, one aspect of impulsivity, are both heritable but that their correlation is mainly driven by family level factors [24]. Similarly, further evidence from genetic studies supports the role of impulsivity as a predisposing factor for overeating and possibly obesity [25]. Else, the association between eating behaviours and ADHD symptoms might be genetically driven. Barker et al. [26] recently observed, that the association between weight, eating disorders and ADHD symptoms might be due to shared genetic liability. They found that ADHD and BMI polygenic risk scores were associated, and both predicted impulsivity and BMI via a cluster of neuroimaging biomarkers. Hence a shared neural substrate might explain the association between these two phenotypes (BMI and ADHD). It still remains to be determined whether eating behaviour lies on this causal pathway.

Although in our previous cross-sectional findings emotional overeating was associated with ADHD symptoms [4], we found that emotional overeating (overeating as a response to negative emotions) was negatively prospectively associated with ADHD symptoms at 6 years. The strong correlation between food responsiveness and emotional overeating may mask an association between emotional overeating and ADHD symptoms, as the analyses are currently adjusted for food responsiveness. Available research has mainly focused on the reverse, suggesting that ADHD may predict disordered eating rather than the other way around [7, 8, 27]. It is also likely that children’s emotional overeating is a manifestation of internalizing symptoms (e.g., symptoms of anxiety and depression) [28] rather than externalizing symptoms and thus it may not necessarily be associated with ADHD symptoms. In addition, we argue that levels of emotional overeating are rather low in early childhood but increase substantially with age, hence this association might become more evident later in life [29].

We found no evidence of an association between eating behaviours at age 4 and later BMI in our study population. Recently Derks et al. were also unable to observe a prospective association between eating behaviours at 4 and BMI at 10 years of age [30]. However, in this study, researchers found the reverse association, that higher BMI at age 4 predicted higher food responsiveness, enjoyment of food and emotional overeating at 10 years. Other studies have shown a positive association between eating behaviours and later weight gain, although associations either attenuated after adjusting for baseline BMI [31–34] or relied mainly on cross-sectional associations with very little prospective data [35]. Inconsistency in findings could be attributed to the bi-directional relationship of BMI with eating behaviours. It is also possible that the association between eating behaviour and weight gain differs across the childhood years, with infancy and early childhood reflecting a critical period for influences of appetite on weight development. Moreover, there is evidence that fat mass may have distinct associations with specific eating behaviours that change over time [33].

Interestingly, we observed that ADHD symptoms at 4 years were not prospectively associated with BMI at 6 years. A recent review of longitudinal studies by Cortese and Tessari supported the causal effect of ADHD on weight gain [6]. The lack of evidence in our study might be due to the low prevalence of obesity in our population at both time points. Given that our previous cross-sectional findings at age 4 did not show any association between ADHD symptoms and BMI [4], and that this association strengthens with age [36, 37] maybe the short period of time between the two follow-up time points (age 4 and 6) was not enough to reflect the effect of ADHD symptoms on BMI. Additionally, available research has shown a differentiation between lean mass and fat mass that we were unable to investigate. A study by Bowling et al. showed that ADHD symptoms at 6 years predicted greater fat mass but not lean mass at the age of 9 years [37].

Notable strengths of the current study include the relatively large sample size, as part of a birth cohort study, allowing us to control for several confounding variables prospectively collected within the cohort. Phenotypes of interest were measured with well-validated instruments, translated and adapted for the Greek population. Furthermore, inclusion of children (almost equal number of boys and girls) from the general population, who do not meet diagnostic criteria for disorder, enables extrapolation of findings to other non-clinical populations. However, extrapolation of the findings might be questioned by the high percentage of non-respondents and their differences with the participating study population.

Despite the strengths, there are some limitations that merit consideration. Even though, various phenotypes were measured in this cohort, other factors, potentially involved in the observed associations were not included; such as impulsivity, parental feeding practices, home environment, and family income [38–41]. Moreover, we were unable to control for any potential confounding effects of condut disorder and maternal BMI. The absence of parental reporting of both ADHD symptoms and eating behaviour can be considered as another limitation. Subjectivity of parental reports may affect the outcome; however, the use of parental reporting in child psychopathology is currently considered the gold standard [42], and behavioural observations in the family home are infeasible in large cohorts. The inclusion of teacher ratings, as an additional measure could have potentially provided a more complete ADHD assessment [43]. In addition, the use of different instruments to assess ADHD symptoms at the two time points may have resulted in differences in the measures of the symptoms and their associations with eating behaviours. Finally, in our study we assessed eating behaviour only at one-time point, at age 4, which precludes the full cross-lagged model.

## Conclusion

In summary, this is the first study that captures the longitudinal association between aberrant eating behaviours and hyperactivity in early childhood. In line with our previous findings, there is evidence that food responsiveness is not only cross-sectionally but also prospectively associated with ADHD symptoms at 6 years of age. In addition, we did not observe any association between ADHD symptoms at 4 years and later BMI or vice versa. We suggest that food responsivenees might be an early sign of possible ADHD symptoms later in life, hence allowing early identification of children who later develop ADHD. Future studies might need to take a broader perspective to better understand if there are causal links between eating behaviours, ADHD symptoms and weight gain, and examine their shared genetic and neurobiological markers.

## Supplementary Information

Below is the link to the electronic supplementary material.

Supplementary file1 (DOCX 28 KB)
