# Supplementary material for: The longitudinal association of eating behaviour and ADHD symptoms in school age children: a follow-up study in the RHEA cohort
Source: Eur Child Adolesc Psychiatry. 2021 Feb 18;31(3):511–7. doi: 10.1007/s00787-021-01720-x (PMC8634555; doi:10.1007/s00787-021-01720-x)
Supplement: Supplementary file 1 — Supplementary file1 (DOCX 28 KB) [file 787_2021_1720_MOESM1_ESM.docx]

**Supplementary Material**

**Table S1. Descriptive statistics of baseline characteristics in the full sample versus participants who had data at both 4 and 6 years of age**

|  | **Baseline, 4 years** | | **Participants with data**  **at 4 and 6 years** | |
| --- | --- | --- | --- | --- |
|  | **N** | **% or Mean± SD** | **N** | **% or Mean± SD** |
| **Maternal age (years)** | **917** | **29.77± 4.99** | **217** | **30.6± 5.13** |
| **Maternal origin, *n (%)*** |  |  |  |  |
| **Greek** | **862** | **93.9** | **263** | **96.34** |
| **Non-Greek** | **56** | **6.10** | **10** | **3.66** |
| **Maternal education, *n (%)*** |  |  |  |  |
| **Low** | **146** | **16.31** | **28** | **10.33** |
| **Medium** | **459** | **51.28** | **130** | **47.97** |
| **High** | **388** | **32.40** | **113** | **41.70** |
| **Child's sex, *n (%)*** |  |  |  |  |
| **Male** | **444** | **47.95** | **143** | **52.19** |
| **Female** | **482** | **52.05** | **131** | **47.81** |

**Table S2.** Standardised path estimates between eating behaviours and ADHD scores at 4 years, CBCL at 6 years and BMI z-scores at both time points using full SEM (n=926).

|  | **Hyperactivity (CBCL)** | | | **Attention Problems (CBCL)** | | | **BMI z-scores 6 years** | | |
| --- | --- | --- | --- | --- | --- | --- | --- | --- | --- |
|  | **β** | **95% CI** | ***P* value** | **β** | **95% CI** | ***P* value** | **β** | **95% CI** | **i value** |
| **CEBQ 4 years** |  |  |  |  |  |  |  |  |  |
| Emotional Overeating | **-0.15** | **(-0.26, -0.04)** | **<0.05** | -0.04 | (-0.16, 0.07) | 0.48 | 0.06 | (-0.01, 0.13) | 0.09 |
| Food Responsiveness | **0.15** | **(0.03, 0.26)** | **<0.05** | 0.10 | (-0.02, 0.22) | 0.11 | -0.04 | (-0.11, 0.03) | 0.30 |
| **ADHD symptoms 4 years** |  |  |  |  |  |  |  |  |  |
| Hyperactivity | **0.37** | **(0.25, 0.49)** | **<0.05** | **0.28** | **(0.15, 0.41)** | **<0.05** | 0.00 | (-0.08, 0.08) | 0.95 |
| Impulsivity | **0.14** | **(0.01, 0.26)** | **<0.05** | **0.17** | **(0.04, 0.30)** | **<0.05** | -0.01 | (-0.08, 0.07) | 0.88 |
| **BMI 4 years** |  |  |  |  |  |  |  |  |  |
| BMI z-scores | 0.00 | (-0.07, 0.08) | 0.94 | 0.01 | (-0.07, 0.09) | 0.81 | **0.82** | **(0.79, 0.86)** | **<0.05** |

Models are adjusted for age, sex, maternal age at birth, maternal education using maximum likelihood for missing variables

**Table S3.** Unstandardized path estimates between eating behaviours and ADHD scores at 4 years, CBCL at 6 years and BMI z-scores at both time points using SEM (n=926), submodel.

|  | **Hyperactivity (CBCL)** | | | **Attention Problems (CBCL)** | | | **BMI z-scores 6 years** | | |
| --- | --- | --- | --- | --- | --- | --- | --- | --- | --- |
|  | **β** | **95% CI** | ***P* value** | **β** | **95% CI** | ***P* value** | **β** | **95% CI** | ***P* value** |
| **CEBQ 4 years** |  |  |  |  |  |  |  |  |  |
| Emotional Overeating | -0.12 | (-0.18, -0.06) | <0.05 |  |  |  |  |  |  |
| Food Responsiveness | 0.06 | (0.01, 0.12) | <0.05 |  |  |  |  |  |  |
| **ADHD symptoms 4 years** |  |  |  |  |  |  |  |  |  |
| Hyperactivity | 0.38 | (0.26, 0.49) | <0.05 | 0.28 | (0.16, 0.41) | <0.05 |  |  |  |
| Impulsivity | 0.14 | (0.02, 0.27) | <0.05 | 0.18 | (0.06, 0.31) | <0.05 |  |  |  |
| **BMI 4 years** |  |  |  |  |  |  |  |  |  |
| BMI z-scores |  |  |  |  |  |  | 0.82 | (0.80, 0.85) | <0.05 |

Models are adjusted for age, sex, maternal age at birth, maternal education using maximum likelihood for missing variables.

**Table S4.** Fit statistics comparing the full model with the submodel (dropping non-significant paths)

|  | **Df*** | **Log likelihood** | **AIC*** | **BIC*** | **Diff log likelihood (df)** | **p-value** |
| --- | --- | --- | --- | --- | --- | --- |
| **Full model** | 41 | -8319.2602 | 16726.520 | 16939.079 |  |  |
| **Constraint model** | 33 | -8322.4346 | 16716.869 | 16890.781 | 6.35 (8) | 0.61 |

* DF: Degrees of freedom; AIC: Akaike’s information criterion; BIC: Bayesian information criterion

**Table S5.** Unstandardized path estimates between eating behaviours and ADHD scores at 4 years, CBCL at 6 years and BMI z-scores at both time points using SEM, for boys (n=482) and girls (n=444).

| **Boys (n=482)** | **Hyperactivity (CBCL)** | | | **Attention Problems (CBCL)** | | | **BMI z-scores 6 years** | | |
| --- | --- | --- | --- | --- | --- | --- | --- | --- | --- |
|  | **β** | **95% CI** | ***P* value** | **β** | **95% CI** | ***P* value** | **β** | **95% CI** | ***P* value** |
| **CEBQ 4 years** |  |  |  |  |  |  |  |  |  |
| Emotional Overeating | -0.71 | (-1.43, -0.001) | 0.05 | -0.22 | (-0.98, 0.52) | 0.55 | 0.09 | (-0.06, 0.06) | 0.99 |
| Food Responsiveness | 0.42 | (-0.06, 0.92) | 0.09 | 0.17 | (-0.35, 0.69) | 0.52 | -0.03 | (-0.14, 0.07) | 0.51 |
| **ADHD Symptoms 4 years** |  |  |  |  |  |  |  |  |  |
| Hyperactivity | **0.17** | **(0.08, 0.26)** | **<0.05** | **0.13** | **(0.04, 0.23)** | **<0.05** | 0.00 | (-0.02, 0.02) | 0.76 |
| Impulsivity | 0.10 | (-0.01, 0.2) | 0.08 | **0.13** | **(0.02, 0.24)** | **<0.05** | 0.00 | (-0.03, 0.02) | 0.54 |
| **BMI 4 years** |  |  |  |  |  |  |  |  |  |
| BMI z-scores | 0.05 | (-0.27, 0.38) | 0.74 | 0.11 | (-0.24, 0.45) | 0.40 | **0.79** | **(0.72, 0.86)** | **<0.05** |
| **Girls (n=444)** |  |  |  |  |  |  |  |  |  |
| **CEBQ 4 years** |  |  |  |  |  |  |  |  |  |
| Emotional Overeating | -0.68 | (-1.43, 0.07) | 0.08 | -0.28 | (-1.05, 0.49) | 0.28 | 0.10 | (-0.09, 0.29) | 0.31 |
| Food Responsiveness | 0.53 | (-0.02, 1.09) | 0.06 | **0.57** | **(0.00, 1.13)** | **0.05** | -0.06 | (-0.20, 0.09) | 0.45 |
| **ADHD Symptoms 4 years** |  |  |  |  |  |  |  |  |  |
| Hyperactivity | **0.22** | **(0.13, 0.32)** | **<0.05** | **0.17** | **(0.07, 0.26)** | **<0.05** | 0.00 | (-0.02, 0.03) | 0.75 |
| Impulsivity | 0.08 | (-0.04, 0.21) | 0.20 | 0.07 | (-0.07, 0.20) | 0.32 | -0.01 | (-0.04, 0.02) | 0.70 |
| **BMI 4 years** |  |  |  |  |  |  |  |  |  |
| BMI z-scores | -0.07 | (-0.42, 0.28) | 0.69 | -0.07 | (-0.42, 0.28) | 0.71 | **0.83** | **(0.75, 0.91)** | **<0.05** |

Models are adjusted for age, sex, maternal age at birth, maternal education using maximum likelihood
